# Spatial and temporal variation in population genetic structure of wild Nile tilapia (*Oreochromis niloticus*) across Africa

**DOI:** 10.1186/1471-2156-12-102

**Published:** 2011-12-09

**Authors:** Etienne Bezault, Patricia Balaresque, Aboubacar Toguyeni, Yves Fermon, Hitoshi Araki, Jean-François Baroiller, Xavier Rognon

**Affiliations:** 1 UMR110 Cirad-Ifremer INTREPID, Montpellier, France; 2 INRA, UMR1313 Génétique animale et biologie intégrative, Jouy-en-Josas, France; 3 Division of Aquatic Ecology, Institute of Ecology & Evolution, University of Bern, Bern, Switzerland; 4 EAWAG, Swiss Federal Institute of Aquatic Science and Technology, Centre of Ecology, Evolution and Biogeochemistry, Department of Fish Ecology and Evolution, 6047 Kastanienbaum, Switzerland; 5 Department of Genetics, University of Leicester, Leicester, UK; 6 CNRS/FRE2960 Laboratoire AMIS (Anthropologie Moléculaire et Imagerie de Synthèse), Toulouse, France; 7 Institut du Développement Rural, Université Polytechnique de Bobo-Dioulasso, Bobo-Dioulasso, Burkina Faso; 8 18 rue J. Richepin, F-91120 Palaiseau, France; 9 AgroParisTech, UMR1313 Génétique animale et biologie intégrative, Paris, France

## Abstract

**Background:**

Reconstructing the evolutionary history of a species is challenging. It often depends not only on the past biogeographic and climatic events but also the contemporary and ecological factors, such as current connectivity and habitat heterogeneity. In fact, these factors might interact with each other and shape the current species distribution. However, to what extent the current population genetic structure reflects the past and the contemporary factors is largely unknown. Here we investigated spatio-temporal genetic structures of Nile tilapia (*Oreochromis niloticus*) populations, across their natural distribution in Africa. While its large biogeographic distribution can cause genetic differentiation at the paleo-biogeographic scales, its restricted dispersal capacity might induce a strong genetic structure at micro-geographic scales.

**Results:**

Using nine microsatellite loci and 350 samples from ten natural populations, we found the highest genetic differentiation among the three ichthyofaunal provinces and regions (Ethiopian, Nilotic and Sudano-Sahelian) (*R*_ST _= 0.38 - 0.69). This result suggests the predominant effect of paleo-geographic events at macro-geographic scale. In addition, intermediate divergences were found between rivers and lakes within the regions, presumably reflecting relatively recent interruptions of gene flow between hydrographic basins (*R_ST _*= 0.24 - 0.32). The lowest differentiations were observed among connected populations within a basin (*R_ST _*= 0.015 in the Volta basin). Comparison of temporal sample series revealed subtle changes in the gene pools in a few generations (*F *= 0 - 0.053). The estimated effective population sizes were 23 - 143 and the estimated migration rate was moderate (m ~ 0.094 - 0.097) in the Volta populations.

**Conclusions:**

This study revealed clear hierarchical patterns of the population genetic structuring of *O. niloticus *in Africa. The effects of paleo-geographic and climatic events were predominant at macro-geographic scale, and the significant effect of geographic connectivity was detected at micro-geographic scale. The estimated effective population size, the moderate level of dispersal and the rapid temporal change in genetic composition might reflect a potential effect of life history strategy on population dynamics. This hypothesis deserves further investigation. The dynamic pattern revealed at micro-geographic and temporal scales appears important from a genetic resource management as well as from a biodiversity conservation point of view.

## Background

The population genetic structure of living organisms is largely shaped by both historical and contemporary gene flow in the species range [1]. Furthermore, the factors structuring the genetic diversity are also able to play a key role in the process of diversification, adaptation and speciation [2]. Identifying the interactions between those factors is crucial to understand the evolutionary history of a species [3]. On the one hand, the extrinsic factors, such as climatic and geological events, shape the large-scale population differentiation pattern [4-6]. On the other hand, both extrinsic (*e.g*. habitat heterogeneity), and intrinsic factors (*e.g*. dispersal capability, mating system and habitat preference) have an impact on gene pool composition at intra-population level or over short time periods [4,7-9]. Species with wide distribution and high dispersal capability are supposed to exhibit genetic differentiation at the paleo-biogeographic scales, with limited micro-geographic structure. On the contrary, species with limited distribution and restricted dispersal capability and/or specific mating behaviour are supposed to exhibit strong genetic structure both at micro-geographic and temporal scales. However, species with a wide distribution area and specific life history traits (*e.g*. restricted dispersal, limited population size) are expected to exhibit more complex genetic diversity pattern.

Cichlid fishes are well known examples of complex population genetic structure among African ichthyofauna. Lake-wide studies have revealed the impact of paleo-historical lake level fluctuations on genetic diversity (*e.g. *[10]). Small scale investigations have revealed the importance of both habitat heterogeneity, ecology and dispersal capability on population differentiation (*e.g. *[11]). To date, however, very few studies have investigated the factors responsible for the population genetic structures both at the micro- and the macro-geographic scales at the same time. They often represent only a relatively restricted paleo-biogeographic scale [12].

The Nile tilapia, *Oreochromis niloticus *(Linnaeus, 1758), is an interesting model-species to study the interactions between intrinsic and extrinsic factors on the structure of the natural populations from a local and temporal to a broader biogeographic scale. This economically important fish has one of the largest natural distributions among African fresh-water fishes, covering the entire Nilo-Sudanian province (from Senegal to Nile basins), the Ethiopian Rift Valley province, the Kivu province, north Tanganyika province (Ruzizi) and the Northern part of the East African Rift Valley. This species shows an exceptional capacity of adaptation, which allowed its colonisation of a wide range of habitats from small forest rivers to large drainage and lakes, as well as alkaline pools with hot springs [13,14]. The description of seven sub-species based on eco-morphology [13] largely reflects their adaptive divergences.

Due to its great interest for aquaculture and fisheries, the Nile tilapia and a few other tilapia species have been introduced outside their natural distributions [14]. Introduced tilapias have become invasive especially in areas originally not containing any tilapiine cichlid, within as well as outside Africa. In area inhabited by congeneric tilapias, on the other hand, they have often led to hybridisation with the local allopatric species [15]. However, between sympatric congeneric species of tilapias, signature of hybridisation has only been reported at evolutionary time scale, not at current ecological time scale. This is especially the case of the ancient mitochondrial introgression from *O. aureus *into *O. niloticus *populations restricted to West Africa, whereas the 2 species are also sympatric in the Nile River. This introgression has certainly happened during the drastic water level fluctuations of the Pleistocene [16]. But neither recent natural hybridisation event nor successful translocation of any allopatric tilapia (*i.e. Oreochromis spp*.) within the natural distribution of *O. niloticus *has been reported so far [15].

As most of the cichlid fish, the Nile tilapia exhibits interesting and complex life history traits, and especially well-developed social behaviour [13,14,17-19]. During reproduction, males show strong territoriality and females provide elaborated parental care (*i.e*. maternal mouth-brooding and guarding) [17]. Because of this reproduction behaviour and substrate affinity, the Nile tilapia is considered a rather sedentary species [14]. In addition, the lekking behaviour of males to attract females for reproduction suggests the existence of a certain level of sexual selection [17,20]. These life history traits are expected to strongly affect the population dynamics of this species, via limited dispersal or reduced effective population size.

From the paleo-geographic point of view, Africa has experienced severe hydrogeographic modifications since the Pleistocene. The East African Rift valley has been subject to many tectonic disruptions of the water basins with inversion of the course of some rivers in the Nile basin (*sensu lato*), whereas the Sudano-Sahelian region experienced dramatic climatic fluctuations with alternating humid and dry phases [21,22]. By their drastic modifications of the extension and connectivity of the different water-basins, all these paleo-geographic and climatic events have undoubtedly affected (1) the distribution of fish species in these ichthyofaunal provinces [23,24] and (2) their population genetic structure.

Previous studies have suggested an influence of paleo-geographic events on the historical distribution of Nile tilapia, based either on morphological traits [13] or on moderately polymorphic molecular markers, *i.e*. allozymes and mtDNA, [16,25-28]. At the opposite, micro-geographic population structure has been reported in lacustrine populations of another tilapiine species, *Sarotherodon melanotheron*, with similar life history strategy, implying non-random mating [29]. However, to date, the relative importance of these paleo-geographic events and the factors influencing the micro-geographic structure of the current populations is not well understood.

In this study, we investigated the spatio-temporal genetic structure of ten natural populations of *O. niloticus*, which cover the main part of the species' natural distribution in Africa. By investigating both spatial and temporal genetic diversity based on nine microsatellite loci, we aimed (1) to understand the current population genetic structure of *O. niloticus *in its natural habitat range in Africa and (2) to evaluate to what extend the population genetic structure was shaped by the paleo-geographic events and the current geographic connectivity at the different spatio-temporal scales. We also discuss potential roles of life history of the species in the species-range population genetic differentiation.

## Results

### Spatial and temporal sampling

Natural populations of *O. niloticus *were sampled from ten geographic sites across Africa to maximise the representation of the biodiversity of the species, naturally spread over the vast Nilo-Sudanian and the Ethiopian ichthyofaunal provinces [23,30]. In order to complement the analyses of population genetic differentiation from macro-geographic to micro-geographic and temporal scales, further sampling was focused along the Volta basin.

**Figure 1:**
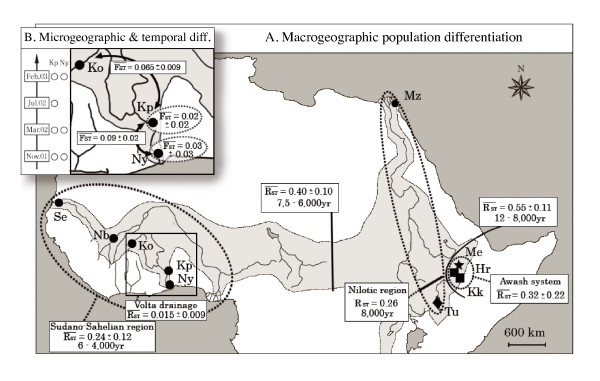
**Population genetic differentiation of Nile tilapia, *Oreochromis niloticus*, across its natural distribution**. Geographic representation of the 10 sampled populations of Nile tilapia within the natural distribution range of the species (shaded area) and summary of the genetic differentiation observed according to the clustering obtained from AMOVA analysis (see Table 4); (A) at macro-geographic level across Africa and (B) zoom on micro-geographic and temporal variability within the Volta basin. Separation times between hydrographical basins are based on paleontological information (see References in the Discussion section). The genetic differentiations are the average pairwise population value observed within (dotted line) or between (solid line) basins or ichthyological provinces and regions; calculations are based on *R*_ST _at Macro-geographic scale, while on *F*_ST_.at micro-geographic and temporal scales, according to the test suggested by Hardy et al. [35] (see Table 3 & 5 respectively). The population symbols refer to the different morphological sub-species: *O. n. niloticus *(black dot), *O. n. vulcani *(black diamond), *O. n. cancellatus *(black square) and *O. n. filoa *(black star) - (see Table 1 for population codes).

Overall, this sample-set of 350 individuals covers six of the larger hydrographic basins naturally inhabited by *O. niloticus*, representing the Nilo-Sudanian ichthyo-province, including Sudano-Sahelian (Niger, Senegal and Volta basins) and Nilotic regions (Turkana and Nile basins) and the Ethiopian Rift Valley province (Awash basin) [23,31]. According to taxonomy [13], this represents four sub-species of *O. niloticus*: *O. n. niloticus *(in West-Africa and Nile) *O. n. vulcani *(in Lake Turkana), *O. n. cancellatus *(in the main part of Awash basin) and *O. n. filoa *(in hot springs of the Awash basin) (Table 1 and Figure 1).

**Table 1:** Population samples information

Sub-species	Ichthyo. Regions	Basin *Lake or River*	Station	GPS location		*Sampling year*	Codes	Sample size
							
				**Lat**.				
	**Ethiopian**	**Awash**					**Aw**	
***O. n. cancellatus***		*Lake Hora*	Debra Zeit	08°45' N	38°59' E	2002	**Hr**	30
***O. n. cancellatus***		*Lake Koka*	Koka	08°24' N	39°01' E	2002	**Kk**	30
***O. n. filoa***		*Lake Metahara*	Abadir	08° 51' N	39°50' E	2002	**Me**	30
***O. n. vulcani***	**Nilotic**	**Lake Turkana**	El Molo Bay	03°37' N	36°02' E	1994	**Tu**	15
***O. n. niloticus***		**Nile**	Lake Manzala	31°17' N	31°59' E	1992	**Mz**	14
***O. n. niloticus***	**Sudano-** **Sahelian**	**Senegal**	Djouj Nat.Park	16°24' N	16°12' W	1991	**Se**	15
***O. n. niloticus***		**Niger**	Bamako	12°38' N	07°60' W	1990	**Nb**	10
		**Volta**					**Vo**	
***O. n. niloticus***		*Kou*	Bama	11°22' N	04°28' W	2004	**Ko**	16
***O. n. niloticus***		*Lake Volta*	Kpandu	06°48' N	00°18' E	Total	**Kp**	103
						*Nov 2001*	**KpN1**	*30*
						*Mar 2002*	**KpM2**	*13*
						*Jul 2002*	**KpJ2**	*30*
						*Feb 2003*	**KpF3**	*30*
***O. n. niloticus***		*River Volta*	Nyinuto	05°56' N	00°41' E	Total	**Ny**	87
						*Nov 2001*	**NyN1**	*28*
						*Mar 2002*	**NyM2**	*30*
						*Feb 2003*	**NyF3**	*29*
							**Total:**	**350**

Taxonomic, bio-geographic and sample size information of the ten populations of Nile tilapia, *Oreochromis niloticus*, studied at both spatial and temporal scales.

Micro-geographic and temporal sampling on the Volta basin was conducted at three sampling localities along the hydrographic basin, of which two were sampled successively multiple times between 2001 and 2003 (Table 1 and Figure 1). They represent 206 individual samples, which were used to estimate temporal variation of population genetic structure as well as effective size of these populations.

### Intra-population diversity

Using nine microsatellite loci, large variation in genetic diversity was observed among populations (Table 2 - for locus by locus information see Additional files 1 &2).

**Table 2:** Genetic diversity per population.

Populations		*A*	*A*r ± SD	H_e _± SD	H_obs _± SD	*F*_IS_		Relatedness			
										
Spatial	*Temporal*							Mr_xy_		Vr_xy_	
**Hr**		2.33	2.15 ± 1.16	0.32 ± 0.28	0.29 ± 0.26	0.089					
**Kk**		2.11	1.68 ± 0.60	0.17 ± 0.21	0.17 ± 0.24	-0.027					
**Me**		2.78	2.31 ± 0.78	0.36 ± 0.23	0.34 ± 0.25	0.063					
**Mz**		5.00	4.75 ± 1.40	0.72 ± 0.10	0.66 ± 0.18	0.090					
**Tu**		5.33	4.94 ± 1.74	0.74 ± 0.11	0.69 ± 0.13	0.070					
**Se**		7.44	6.30 ± 3.41	0.67 ± 0.27	0.63 ± 0.29	0.058					
**Nb**		3.00	3.00 ± 1.41	0.42 ± 0.24	0.39 ± 0.28	0.076					
**Ko**		6.33	5.30 ± 1.77	0.65 ± 0.18	0.64 ± 0.19	0.020					
**Kp**		9.89	5.79 ± 1.94	0.69 ± 0.19	0.58 ± 0.15	0.158	***	-0.01		0.22	
	
	*KpN1*	*6.44*	*5.12 ± 2.08*	*0.64 ± 0.24*	*0.54 ± 0.21*	*1.154*	***	*-0.03*	**	*0.24*	
	*KpM2*	*4.56*	*4.49 ± 1.70*	*0.69 ± 0.17*	*0.63 ± 0.26*	*0.086*		*-0.08*		*0.29*	*
	*KpJ2*	*7.11*	*5.40 ± 1.79*	*0.66 ± 0.19*	*0.55 ± 0.15*	*0.180*	***	*-0.03*		*0.21*	
	*KpF3*	*7.56*	*5.90 ± 2.17*	*0.71 ± 0.17*	*0.65 ± 0.19*	*0.090*	*	*-0.04*		*0.21*	
**Ny**		8.56	5.37 ± 2.51	0.64 ± 0.19	0.55 ± 0.24	0.149	***	-0.04	***	0.35	
	
	*NyN1*	*6.57*	*5.35 ± 2.52*	*0.67 ± 0.16*	*0.52 ± 0.37*	*0.230*	***	*-0.07*		*0.40*	
	*NyM2*	*5.25*	*4.19 ± 2.10*	*0.55 ± 0.20*	*0.46 ± 0.27*	*0.156*	***	*-0.04*		*0.27*	**
	*NyF3*	*6.22*	*4.84 ± 2.73*	*0.60 ± 0.25*	*0.53 ± 0.26*	*0.111*	*	*-0.04*	*	*0.27*	**

Genetic diversity and allelic richness observed and estimated from rarefaction algorithm calculated over the nine loci for the ten study populations and their temporal samples (codes are given in Table 1), as well as level of relatedness r_xy _[63], estimated based on mean (Mr_xy_) and variance (Vr_xy_) of the distribution of pairwise relatedness index, calculated for each temporal samples from each location and across temporal samples for each location (see main text for additional details)

*A *= number of alleles observed per population; *A*r = allelic richness per population based on rarefaction algorithm; *H*_e _= non-biased expected heterozygosity; *H*_obs _= observed heterozygosity (with standard deviations = SD); the *F*_IS _= fixation index values with their significances after sequential Bonferroni correction; Test significance: *P < 0.05, ** *P *< 0.01, *** *P *< 0.001.

The populations of the Awash basin showed the lower gene diversity based on both allelic richness (*A*r = 1.68 - 2.31) and heterozygosity (*H*_o _= 0.17 - 0.34). All other Nilotic and Sahelo-Sudanian populations presented an overall higher level of genetic diversity (*A*r = 4.75 - 6.30; *H*_o _= 0.55 - 0.69), except for Niger revealing an intermediate level of diversity (*A*r = 3.00; *H*_o _= 0.42).

Departure from Hardy-Weinberg equilibrium was detected for 5 of the 10 geographic populations (*i.e*. Hora, Metahara, Turkana, Kpandu and Nyinuto). The test remained significant after Bonferroni correction for the two populations from the Volta basin constituted of temporal sub-sampling (Kpandu, Kp, and Nyiinuto, Ny). Both populations showed significantly positive *F*_IS _values, suggesting a deficit of observed heterozygotes (*P *< 0.001, Table 2). This result was consistent with a temporal Wahlund effect at the population level when pooling slightly differentiated temporal samples (*F*_ST _= 0 - 0.053 - Table 3 and see below). However, significantly positive *F*_IS _was also found in all but one temporal sample from these two populations (*i.e*. except KpM2 with the smallest sample size). The deficit of heterozygotes could be due to either i) null alleles, ii) a spatial Wahlund effect, or iii) non-random mating. After exclusion of the three loci potentially possessing null alleles (*see detail in Methods section*), however, four samples (*i.e*. two in Kpandu and two in Nynuto) still exhibited significant positive *F*_IS _values. Thus, null alleles are not the main cause of the disequilibrium. The possibility of the spatial Wahlund effect, namely artificial substructure from the pool of different fishing catches within the temporal samples, was tested using AMOVA. No significant differentiation among seine catches was detected (*P *= 0.386; *data not shown*), suggesting the absence of Wahlund effect at this level. To investigate a possibility of non-random mating within population and cohorts, we analysed the level of relatedness among individuals at both i) site and ii) temporal levels, and compared the distribution of the observed pairwise relatedness index (r_xy_) to simulated distribution expected under panmixia. At the site level, Nyinuto population presented an overall lower level of relatedness (Mr_xy_, *P < 0.001*) than expected under panmixia, however the population from Kpandu did not differ from the null expectation. Furthermore, the majority of the temporal samples showed significant difference in level of relatedness than expected in a randomly mating population in both populations: two temporal samples showed higher average pairwise relatedness (*i.e*. KpN1 & NyF3) and one showed a higher variance of relatedness distribution (*i.e*. KpM2), while one sample showed a lower variance of relatedness distribution (*i.e*. NyM2 - Table 2). Thus, though the three possible causes of the deficit of heterozygotes are not mutually exclusive, non-random mating within each temporal sample is seemingly contributing to the observed pattern most.

**Table 3:** Matrix of macro-geographic population differentiation based on R*_ST _*and F*_ST _*estimators

	Hr	Kk	Me	Mz	Tu	Se	Nb	Ko	Kp	Ny
**Hr**		0.427***	0.072**	0.601***	0.472***	0.713***	0.636***	0.693***	0.587**	0.660***
**Kk**	0.296***		0.473***	0.696***	0.582***	0.833***	0.787***	0.816***	0.709***	0.770***
**Me**	0.059**	0.287***		0.553***	0.377***	0.692***	0.599***	0.671***	0.573***	0.632***
**Mz**	0.451***	0.553***	0.425***		0.259***	0.384***	0.247***	0.397***	0.425***	0.424***
**Tu**	0.442***	0.549***	0.416***	0.115***		0.431***	0.209***	0.469***	0.520***	0.517***
**Se**	0.489***	0.596***	0.467***	0.225***	0.229***		0.217***	0.152***	0.133**	0.101**
**Nb**	0.603***	0.723***	0.585***	0.326***	0.331***	0.243***		0.377***	0.374***	0.349***
**Ko**	0.507***	0.607***	0.481***	0.222***	0.214***	0.097***	0.306***		0.0249*ns*	0.0073*ns*
**Kp**	0.404***	0.456***	0.392***	0.216***	0.221***	0.054**	0.246***	0.054**		0.0142*ns*
**Ny**	0.455***	0.524***	0.440***	0.243***	0.224***	0.101***	0.261***	0.095**	0.078**	

Pairwise *R*_ST _(above diagonal) and *F*_ST _(below diagonal) for the ten spatial populations studied, with corresponding level of significance (based on 10,000 replicates). The significance of the test of Hardy [35] (*P *< 0.001) suggests that *R*_ST _is more appropriate to describe genetic differentiation among these populations than *F*_ST_.

**Figure 2:**
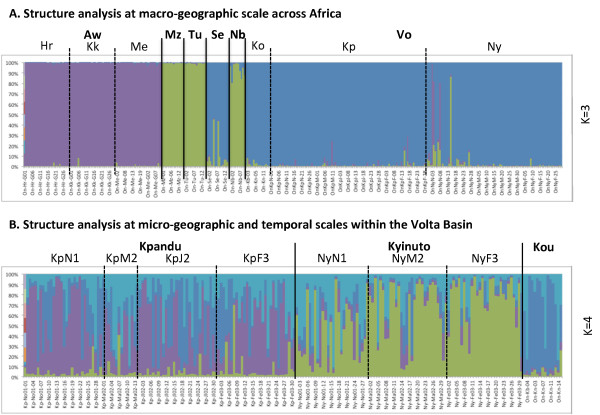
**Population structure at macro-geographic, micro-geographic and temporal levels**. Individual-based Bayesian clustering using STRUCTURE v.2.3.1[32,33] to assess the genetic population structure at both **A) **macro-geographic and **B) **micro-geographic and temporal scales. For each scale, the number of genetic clusters (K), which was best supported by the procedure proposed by Evanno et al. (ΔK) [34] was represented (*i.e*. K = 3 for A and K = 4 for B). Different colours represent different genetic clusters in each figure. Code representing populations and hydrographic basins are given above graphs (see Table 1 for details). Results for different K are shown in Additional file 3.

A Bayesian-based population assignment analysis using STRUCTURE[32,33] revealed that more than 91% of the individuals belong to a single genetic cluster with a posterior probability of > 95% (Figure 2a), further reflecting expected biogeographic affinities among populations (see below); only 3.7% of individuals (13 out of 350) showed with an assignment > 10% posterior probability to another genetic cluster, suggesting little impact of translocation or recent hybridisation in the studied populations.

### Macro-geographic populations structure & differentiation

**Figure 3:**
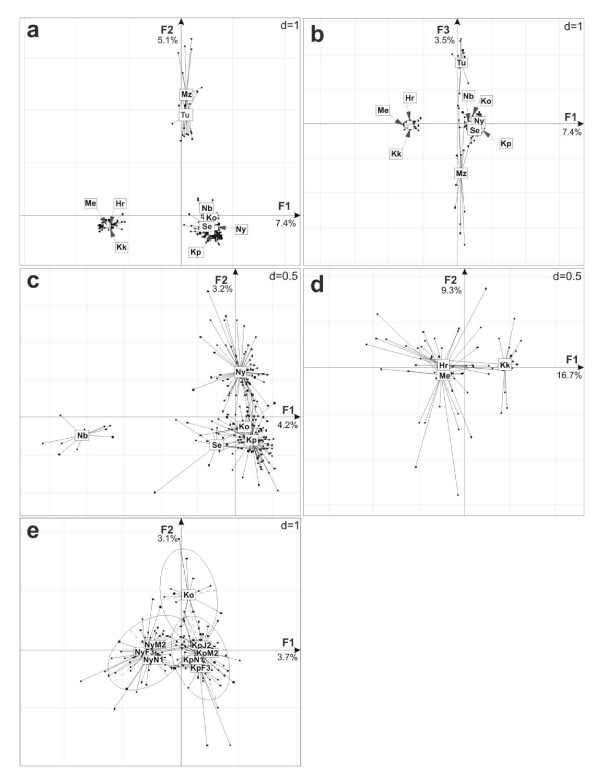
**Hierarchical spatio-temporal population clustering**. Factorial correspondence analyses based on genotype of *O. niloticus *samples at successive hierarchical spatial and temporal levels, depicting the best population clustering. For each population a star representation is used: every individual is linked with a line to the barycentre of its own population (labelled using sample code according to Table 1). At macro-geographic scale, we performed the projection of the 10 populations representing the overall biogeographic region studied (*i.e*. Ethiopian Rift Valley province, Nilotic, Sudano-Sahelian regions) on factorial planes F1 × F2 (**a**) and F1 × F3 (**b**), the projection of the Sudano-Sahelian region (*i.e*. West-African populations: Niger, Senegal and Volta basin populations) on factorial plane F1 × F2 (**c**), the projection of the Ethiopian Rift Valley province populations (*i.e*. Awash basin) on factorial plane F1 × F2(**d**). For the micro-geographic and temporal scales analysis, we performed the projection of the samples from the Volta basin including Kou, Kpandu and Nyinuto populations (temporal sample series are represented by stars and the area of each geographic population is represented by a 95% confidence ellipse), on factorial plane F1 × F2 (**e**). For each correspondence analysis, the percentage of variation represented by each factorial axis is given aside of its label.

At macro-geographic level, factorial correspondence analysis (FCA) discriminated three distinct groups of populations along the first two factorial axes (F1 & F2; Figure 3a): the first group corresponded to the three Ethiopian populations, Hora, Koka and Metahara, the second group to the five populations from the Sudano-Sahelian region, Niger, Senegal and Volta (Kou, Kpandu and Nyinuto), and the third group to the Nilotic populations of Nile and Turkana, which were further separated by the third factorial axis (F3; Figure 3b). This population clustering is reflecting the biogeographic separations rather than taxonomic description. The STRUCTURE analysis, for the best supported number of clusters (K = 3) according to the Evanno correction [34], revealed a similar clustering pattern between populations from the Ethiopian province and, Nilotic and Sudano-Sahelian regions (Figure 2a). However, the population from Niger was predominantly associated with the Nilotic group rather than the Sudano-Sahelian one. Nevertheless, the assignment of the Niger population become shared between Nilotic and Sudano-Sahelian groups and then predominantly associated with the Sudano-Sahelian group, at K = 4 and higher respectively (see Additional file 3). These results point out a slightly more central position of the Niger basin within the Sahelian region, compared to the Nilotic region (also supported by genetic differentiation - see below). Furthermore the separation of the two populations from the Nilotic region was also observed when K was higher than the optimal K = 4 (see Additional file 3).

The significance (*P *< 0.001) of the test suggested by Hardy et al. [35] revealed that SMM-based estimator (*R_ST_*, see Methods) is the most pertinent to describe genetic differentiation at macro-geographic scale The highest population differentiations were found between regions (Table 3), especially between Ethiopian and Sudano-Sahelian populations ($\bar{R_{\text{ST}}}$ = 0.695 ± 0.08), while the Nilotic populations exhibit a high degree of genetic differentiation from either Ethiopian or Sudano-Sahelian populations ($\bar{R_{\text{ST}}}$ = 0.55 ± 0.11 and $\bar{R_{\text{ST}}}$ = 0.40 ± 0.10, respectively; Figure 1). The hierarchical AMOVA conducted with populations clustered by biogeographic sub-regions revealed that 49.4% of the genetic variance was actually distributed between Ethiopian province and, Nilotic and Sudano-Sahelian regions, whereas 6.7% was distributed among population within regions, both with very high significance (*P *< 0.001). The alternative AMOVA model, with populations clustered according to taxonomy, showed comparatively less fitted variance partitioning, with lower percentage of variance explained by the clustering factor (47.3%). Although this result was highly significant, a larger part of the genetic variance was attributed to the between-populations within group component (9.1%). When samples were clustered by hydrographic basins, 49.8% of the genetic variability was partitioned among groups, and 3.5% was detected among populations within basins (*R_SC _*= 0.069, *P *< 0.001), revealing intra-basin heterogeneity.

Analyses conducted at the biogeographic region level reveal variable level of differentiation, as 61.2% of the genetic variance of the system is contained within populations of the Ethiopian Rift Valley province (n = 3), while 90.6% for the Sudano-Sahelian region (n = 5). Within Ethiopian Rift valley province, the multivariate analysis widely separates the three populations across the first factorial plane (F1 × F2; Figure 3d), confirmed by an average differentiation of $\bar{R_{\text{ST}}}$ = 0.32 ± 0.22 (Figure 1): Hora and Metahara populations being clearly overlapping, with the lowest pairwise differentiation (*R_ST _*= 0.072, *P *< 0.01), while Koka appeared more isolated, especially from Metahara (0.473, *P *< 0.001). Within the Sudano-Sahelian region, 24.8% of the variance was observed between hydrographical basins (Table 4). Multivariate analysis and pairwise genetic differentiation reveal that a large part of this differentiation is due to the separation of the Niger from the remaining basins (F1; Figure 3c), $\bar{R_{\text{ST}}}$ = 0.264 ± 0.001 (Figure 1). The among-population, within-group differentiation was low and not significant (*R*_SC _= 0.002) indicating certain homogeneity within basins. Within the Volta basin, almost all of the genetic variance (~100%) was found within populations with very little differentiation between sample sites ($\bar{R_{\text{ST}}}$ = 0.015 ± 0.009; Figure 1). This result was concordant with the fact that populations within the Volta basin are still connected.

**Table 4:** Hierarchical partitioning of the genetic variance.

**Stat**.		Variance components (%)			Φ **Statistics**		
**Estim**.	Grouping	Within pops	Among groups	Among pops within groups	Φ**_ST_**	Φ**_CT_**	Φ**_SC_**
	**Macro-geographic scale**						
	All populations in one group	54.69		45.31	0.453 ***		
	All populations grouped by region ^0^	43.90	49.42	6.68	0.561 ***	0.494 ***	0.132 ***
	All populations grouped by basin ^1^	46.74	49.78	3.48	0.533 ***	0.498 ***	0.069 ***
	All populations grouped by taxonomy ^2^	43.72	47.14	9.14	0.563 ***	0.471 ***	0.173 ***
***R*_ST_**	within Sudano-Sahelian region	90.61		9.39	0.090 ***		
	within Sudano-Sahel. grouped by basin^3^	75.03	24.79	0.18	0.250 ***	0.248 ^ns^	0.002 ^ns^
	within Ethiopian region (Awash)	61.16		38.84	0.388 ***		
	within Nilotic region (Turkana & Nile)	73.05		26.95	0.269 ***		
	within Volta basin	100.45		-0.45	0.004 ^ns^		

	**Micro-geographic & temporal scale**						
	within Volta basin	93.67		6.33	0.063 ***		
***F*_ST_**	within Volta grouped by site ^4^	91.56	6.48	1.96	0.084 ***	0.065 **	0.021 ***
	within Kpandu (all temporal samples)	98.71		1.29	0.013 *		
	within Nyinuto (all temporal samples)	92.61		7.39	0.074 **		

Hierarchical analysis of molecular variance (AMOVA) calculated based on (1) *R*-Statistics for the macro-geographic scale and on (2) *F*-Statistics for the micro-geographic and temporal scales, considering the results of the test suggested by Hardy *et al. *[35]; the populations grouping reflects ichthyological provinces and regions, hybrographic basins, and taxonomy status (cf. Table 1).

^0 ^3 groups: (Ethiopian: Hr, Kk, Me), (Nilotic: Mz, Tu), (Sudano-Sahelian: Se, Nb, Ko, Kp, Ny)

^1 ^6 groups: (Awash: Hr, Kk, Me), (Nile: Mz), (Turkana: Tu), (Senegal: Se), (Niger: Nb), (Volta: Ko, Kp, Ny)

^2 ^4 groups: (*O.n.cancellatus*: Hr, Kk), (*O.n.filoa*: Me), (*O.n.vulcani*: Tu), (*O.n.niloticus*: Mz, Se, Nb, Ko, Kp, Ny)

^3 ^3 groups: (Senegal: Se), (Niger: Nb), (Volta: Ko, Kp, Ny)

^4 ^3 groups: (Ko), (Kp: KpN1, KpM2, KpJ2, KpF3), (Ny: NyN1, NyM2, NyF3)

Test significance: ns = non-significant, * *P *< 0.05, ** *P *< 0.01, *** *P *< 0.001.

Overall, these results demonstrate that ichthyofaunal provinces definition and hydrographical basins boundaries are the two major structuring factors of *O. niloticus *populations at large geographic scales.

### Micro-geographic and temporal comparisons

The analysis of micro-geographic and temporal variations has been conducted along the Volta basin. Based on factorial correspondence analysis (Figure 3e), three groups visibly emerged in the first factorial plan (F1 × F2), clustering the individuals according to their different geographic origins, Kou, Kpandu and Nyinuto. Temporal samples from Kpandu and Nyinuto were very close to each other within each location. Similarly, the STRUCTURE analysis reveals an optimal number of four clusters, representing preferentially the three different geographic locations, without strict discrimination (Figure 2b). The fourth genetic cluster was relatively equally distributed across all sites. No clear differentiation among temporal samples was observed at K = 4 or higher number of cluster (see Additional file 3).

This pattern was confirmed by the partition of the genetic variance and pairwise population differentiations based on *F*_ST _(*i.e*. the non-significance of the test proposed by Hardy et al [35] suggesting that IAM-based estimator is the most suitable to depict genetic differentiation at this scale - Table 5, see also Methods).

**Table 5:** Matrix of micro-geographic and temporal population differentiation based on F*_ST _*estimators.

	Ko	KpN1	KpM2	KpJ2	KpF3	NyN1	NyM2	NyF3
**Ko**								
**KpN1**	0.068**							
**KpM2**	0.069***	0.029*						
**KpJ2**	0.052**	0.016*ns*	-0.015*ns*					
**KpF3**	0.072***	0.024*	0.038**	0.030*				
**NyN1**	0.101***	0.103***	0.060***	0.092***	0.053**			
**NyM2**	0.116***	0.130***	0.118***	0.106***	0.105***	0.045**		
**NyF3**	0.106***	0.099***	0.100***	0.103***	0.080***	0.053***	-0.0037*ns*	

**F*_ST _***(below diagonal) for the eight spatio-temporal samples from the 3 geographic populations studied along the Volta basin, with corresponding level of significance (based on 10,000 replicates). The non-significance of the test of Hardy [35] suggests that **F*_ST _***is more appropriate to describe genetic differentiation among these populations than **R*_ST_***.

Test significance: ns = non-significant, * *P *< 0.05, ** *P *< 0.01, *** *P *< 0.001.

S; below diagonal) and associated level of significance (above diagonal).

The highest genetic differentiation was found between geographic sites in the Volta basin (*F*_ST _= 0.05 - 0.13, $\bar{F_{\text{ST}}}$ = 0.09 ± 0.02; Figure 1), representing 6.5% of the genetic variance of the system (Table 4). The genetic differentiations within temporal series in Kpandu (*F *= 0 - 0.04, $\bar{F}$ = 0.02 ± 0.02) and Nyinuto (*F *= 0 - 0.05, $\bar{F}$ = 0.03 ± 0.03) were lower than observed between sample sites, but yet significant (Table 5). Partition of the genetic variance revealed that 2.1% of the genetic variance was due to genetic differentiation in time (across both Kpandu and Nyinuto; Table 4). To examine whether the genetic differentiation between temporal series was due to the effect of one sample site or both, we performed separate AMOVA on Kpandu and Nyinuto samples, respectively. Interestingly, we found that genetic variance due to among temporal series variability was significant for both sites: 1.3% for Kpandu (*F*_ST _= 0.013, *P *< 0.05) and 7.4% for Nyinuto (*F*_ST _= 0.074, *P *< 0.01).

This demonstrates that subtle temporal changes of gene pool composition need to be considered in population structuring of *O. niloticus *at micro-geographic scale.

### Effective population size and immigration rate

The effective population size (*N_e_*) was estimated based on two focal populations in the Volta basin (Kpandu & Nyinuto). We used three different methods, based either on temporal variations of allele frequency within a population, compared the global gene pool of the basin [36], or on the level of linkage disequilibrium (LD-*Ne*) within population [37]. Two temporal methods were used in this study. The LD-*Ne *and the first temporal method (*Ne*_CLOSED_) assume absence of immigration to estimate *N_e _*[38]. The second temporal method (*Ne*_open_) relaxes this assumption and assume connectivity between populations to jointly estimate *N_e _*and immigration rate (*m*) [36] (Table 6). Point estimates of *N_e _*ranged between 31-143 for Kp and 23-34 for Ny. While different methods provided different confidence intervals (*N_e_*: from 21-53 to 70-817 for Kp, and from 17-33 to 23-55 for Ny), real *N_e _*would likely be in or around the above ranges (see discussion in [39]). Estimates of the immigration rate (*m*) were similar for both populations (*m *~ 0.097 for Kp, and *m *~ 0.094 for Ny).

**Table 6:** Effective population size and dispersal rate.

Estimator	***Ne***_**OPEN **_**&*m***					***Ne***_**CLOSED**_		LD-*Ne*	
	
Population	Source	*Ne*	95%CI	*m*	95%CI	*Ne*	95%CI	*Ne*	95%CI
**Kpandu**	*Ko+Ny*	31	21-53	0.097	0.053-0.160	143	70-817	61	37-142
**Nyinuto**	*Ko+Kp*	27	19-44	0.094	0.052-0.157	34	23-55	23	17-33

Estimates of effective population size (*Ne*) and dispersal rate (*m*) from temporal samples of two populations from the Volta basin using three different methods: (1) maximum likelihood temporal method to jointly estimate *Ne*_open _and migration *m *[36], (2) pseudo-likelihood temporal method to estimate *Ne*_closed _[38], and (3) point-estimate Linkage-Disequilibrium based method LD-*Ne *[37] averaged over the different temporal samples (KpM2 (n = 13) was excluded from the calculations).

## Discussion

### Phylogeography of the Nile tilapia: past history and current genetic structure at macro-geographic scale

From the Miocene (*i.e*. 20-5 My BP) to the Pleistocene (*i.e*. 2 My to 10,000 yrs BP), numerous paleo-geographic (*e.g*. causing disruption of river drainages) and paleo-climatic events (*e.g*. humid and dry phase cycles), by changing the connectivity between the different hydrographical systems, have deeply affected African ichthyofauna [22-24,40]. Biogeographic data and fossil records suggest a Nilotic origin of the Nile tilapia (Upper Pliocene ~ 5-2 My BP [13]) - compatible with allozyme data [25]. The present study identified three genetic clusters of Nile tilapia populations corresponding to the major biogeographic subdivisions: (1) the Ethiopian Rift Valley ichthyofaunal province (*i.e*. the Awash system), (2) the Nilotic region (*sensu lato*, comprising the presently separated but previously connected Nile River and Turkana basin) and (3) the Sudano-Sahelian region (*i.e*. West-African basins) (Figure 1), the two later belonging to the Nilo-Sudanian ichthyofaunal province [23]. The comparison of the AMOVA's results revealed a first differentiation of population between ichthyofaunal regions (49%), as also depicted by STRUCTURE analysis. Therein genetic variance was further partitioned between hydrographic basins (1-3% of genetic variance between basins independently of ichthyological regions), and further remaining significant molecular variance among populations within basins (3.5%). This pattern is consistent with those observed in the few other fish species widespread across the Nilo-Sudanian area and studied by genetic markers, *Tilapia zillii *[25], *Oreochromis aureus *[16] and *Clarias gariepinus *[41]. In particular, these studies also emphasise the deep split between the Nile and the Sudano-Sahelian populations. The westward spread of this species from the Nile to Sudano-Sahelian rivers has probably occurred during the Pleistocene [13,14], presumably facing the raise of mega-paleo-lakes and drainage connectivity in the Saharan region [21,24,40].

The Ethiopian populations exhibited the lowest levels of polymorphism within each population and the largest genetic divergence from the other ones (Tables 2). The isolation of the Awash from the Nile system occurred by tectonic disruption of this river basin about 12,000-8,000 years ago [23], which can explain the substantial genetic divergence between these two basins ($\bar{R_{\text{ST}}}$ = 0.55 ± 0.011 - Figure 1). The low genetic diversity in all three Awash populations (Table 2) and rather high differentiation ($\bar{R_{\text{ST}}}$ = 0.32 ± 0.22 - Figure 1) among them suggest strong effects of genetic drift within this "islands system", which exhibits an extensive level of fragmentation between the Awash River and several relatively isolated highland lakes. Following Trewavas [13], two different morphological sub-species coexist in the Awash basin: *O. n. cancellatus *in the river and cold-water lakes (*e.g*. Lakes Hora and Koka), and *O. n. filoa *endemic to the hot spring areas (*e.g*. Lake Metahara). The lower genetic distance between Hora and Metahara (*R*_ST _= 0.07) compared to Koka (*R*_ST _= 0.43 and 0.47 respectively) does not reflect their taxonomic status (*i.e*. paraphyly of *O. n. cancellatus*). In addition, the description of *O. n. filoa *is mostly based on the reduced number of some meristic characters (*i.e*. number of vertebra, scales), which might be subject to developmental plasticity [13]. These results, as well as the similar reproduction pattern and the absence of post-zygotic barrier [42,43] support the hypothesis that *O. n. filoa *and *O. n. cancellatus *are two ecotypes, rather than distinct subspecies. However, further specific experiments, especially according to environment-induced developmental plasticity and mate choice preference, should be conducted to confirm this hypothesis.

In the Nilotic system *sensu lato*, disconnection occurred between the Nile river and the currently endorheic Lake Turkana basin about 8,000 years ago [21,44]. Individuals from these two geographically distant populations have been previously identified as two distinct subspecies, *O. n. niloticus *and *O. n. vulcani *respectively [13]. However, compared to the large geographic distance and separation time existing between these two populations, the genetic differentiation was expected to be higher than observed (*R*_ST _= 0.26 - Figure 1) - with respect to other values observed in the present study. Connections between Lake Turkana and the Nile River have probably existed at least periodically until a few thousand years ago [44], as it seems to be reflected by nuclear and mtDNA data [16,25-27].

The Sudano-Sahelian basins were isolated from the Nile River by multiple paleo-geographic events between 7,500 and 6,000 years ago [21]. Although morphological analyses have clustered populations from the Nile River with those of the Sudano-Sahelian basins [13], previous allozyme data [25,27] and this study ($\bar{R_{\text{ST}}}$ = 0.38 ± 0.07) revealed a clear disconnection between these two biogeographic entities. This independent history is reinforced by the differential pattern of ancient introgression of mtDNA from *O. aureus *to *O. niloticus *involving the whole West African area, but not reported in any Nilotic population [16].

The Chad-Chari system appears to have played a key role between the Nilotic and Sudanian biogeographic entities, providing an unidirectional connectivity for the ichthyofauna from the Nile to the West African drainages, via the Niger, during the periods of growth of the mega-paleo-Lake Chad [21,24,40,45]. The West African populations are the most geographically widespread, but also the least morphologically diversified [13]. They also exhibited the lowest level of genetic differentiation between basins ($\bar{R_{\text{ST}}}$ = 0.24 ± 0.12 - Figure 1). Such a pattern might be explained by the persistence of episodic connections among Senegal, Niger and Volta headwater between 6,000 and 4,500 years ago [22]. Despite these recent gene flow events, all the Sudano-Sahelian populations could be strictly distinguished using microsatellites, even for populations at an intra-basin level, such as for the Volta River, contrary to the previous results obtained using allozyme or mitochondrial data [25].

As expected, no signature of anthropogenic impact on population differentiation was identified in this study. Paleo-geographic and climatic events appear to be important factors for the current pattern of the genetic structure of Nile tilapia populations within its natural distribution across Africa.

### Microgeography and temporal variation

Within the Volta basin, most of the genetic variability was distributed within populations (93.7%), from the upstream Kou River, to the central Lake Volta (Kpandu) and downstream Volta River (Nyinuto). This suggests their recent differentiation and/or non-interrupted gene flow. The genetic differentiation within temporal series in Kpandu ($\bar{F}$**= **0.02 ± 0.02) and Nyinuto ($\bar{F}$**= **0.03 ± 0.03) was lower than between sample sites ($\bar{F_{\text{ST}}}$ = 0.09 ± 0.02; Figure 1), but still significant and representing more than 2% of the genetic variance of the system. These results suggest a geographic differentiation between populations, and a quick turnover of the gene pools within population within only a few generations.

Our estimates of the effective population size from three different approaches consistently showed larger *N_e _*in Kpandu than in Nyinuto (Table 6). The estimated migration rate (*m*), obtained jointly with *Ne*_open _[36], provided similar values for both populations. Although the size of each sample for these estimates was limited (n = 28 - 30), the congruence of the different *N_e _*estimates together with relatively small confidence intervals indicates a good coherence and reliability of our estimations [39,46]. The population of Kpandu, located at the shore of Lake Volta (one of the largest artificial lakes in the world), is believed to have a large census size and/or well connected to neighbouring populations. In contrast, the population of Nyinuto, located at the downstream limit of Nile tilapia distribution along the Volta River, is likely to have a more limited size and to be easily subject to fluctuations (*e.g*. variation of water level and salinity - E. Bezault *pers. obs*.), and/or more isolated, than a central basin population. Despite the potential limit associated to our sampling strategy, our *N_e _*estimates consistently showed relatively small effective population size of the natural Nile tilapia populations relative to expected census population size (*i.e*. fisheries records expressed in millions of tonnes per year for the Volta basin; E. K. Abban, *pers. comm*.) and a non-negligible dispersal rate.

The hypothesis of the effect of specific social and reproductive behaviour of the Nile tilapia emerges from the positive *F*_IS _observed in both populations and the significant relatedness detected in some temporal samples (*i.e*. 3 out of the 4 temporal samples with significant relatedness showing either a higher mean or variance relatedness distribution, revealing respectively overall related individuals or subgroups of related individuals). A shoaling behaviour involving relatives could for instance explain such a pattern. This behaviour has been observed in other populations of *O. niloticus *[47,48] as well as in another tilapia species, *Sarotherodon melanotheron *[29,47]. These authors suggested this pattern to be due to direct inbreeding, implying preferential reproduction between individuals related among themselves, as observed in other cichlid species [49]. Furthermore, it has been claimed that this behaviour appears preferentially in open-water habitats, and especially in lagoon systems [29]. In the present study, the pattern of inbreeding observed in both lake and river habitats cannot be totally explained by shoaling behaviour among relatives, due also to the absence of sub-structure related to seine net catches (*i.e*. potential shoal) within temporal samples. A more direct effect of a non-panmictic reproductive system should be considered. Polygynandric mating system, generally exhibited, by maternal mouthbrooding cichlids, as *O. niloticus*, is generally associated with sexual selection and pronounced between-individual variations in reproduction success [17,20]. Such variance of fitness has been reported for male Nile tilapia but only in captivity [50]. The rapid turnover in gene pool and the restricted effective population sizes, observed in our study, could be explained by the differential contribution of breeders to within and between cohorts over generations.

Furthermore the non-negligible dispersal rate detected in the present study opens up new research perspectives on cichlids. While sophisticated reproduction strategy and extended parental care are expected to increase territoriality and consequently prevent adult migration, our results suggest that the exceptional adaptive plasticity and riverine affinity of the tilapia could also be seen in its dispersal leading to larger migration rate than more specialised (stenotypic) cichlid species from East African Great Lakes [11,20,51]. This hypothesis is corroborated by the population genetic analysis of the riverine haplochromines generalist, *Pseudocrenilabrus multicolour victoriae*, presenting relatively large distribution area with geographic structure being the main driver of population structure and gene-flow [12]. It has also been demonstrated in this species important change of gene pool composition at short temporal scale (*i.e*. 2 years), which has been associated with drastic fluctuation of hydrographic regime [52]. Larger estimates of effective population size (including higher confidence interval reaching infinity) have been obtained for *P. m. victoriae *in this study compared to effective population size estimated here for *O. niloticus*. Unfortunately no census population size estimate for any of these species is available to effectively compare the *N_e_*/*N *ratio, however, in the case of *P. m. victoriae *it cannot be ruled out that the larger effective population size observed is influenced by flooding regime.

## Conclusions

The distribution and pattern of genetic diversity of a species over its natural range is the result of multiple and interlinked factors, which acted through space and time. For the Nile tilapia, *O. niloticus*, the genetic pattern observed from large geographic to temporal scales first reflects the hierarchical impact of paleo-geographic and climatic events as well as the impact of hydrographic connectivity on population genetic structure. Furthermore, the hierarchical pattern of genetic differentiation seems to reflect only bio-geographic events, without revealing any signature of anthropogenic impact. Compared to the most studied, highly radiating cichlid lineages, the morphologically and ecologically generalist species, as especially the tilapiines, [13,19], might have distinct population dynamic characteristics. The relatively small effective population size associated with a moderate dispersal and the rapid change in gene pool composition in only a few generations tend to reinforce our current knowledge about the biology of this species, and they might reflect a potential impact of natural history strategy on population dynamics. Further investigations are needed to confirm this hypothesis. In addition, more research emphasis should be put on micro-geographic population structures to evaluate a more precise relationship between census and effective population size, the extent of dispersal among the natural population of Nile tilapia, as well as the balance between selection and gene-flow across different habitats. Furthermore, the possibility that Nile tilapia populations may be organised in meta-populations within larger and more connected systems (like within the Lake Volta) should be further investigated, from a genetic resource as well as from a conservation point of view.

## Methods

### Sampling and analysis strategy

We collected 350 samples of *O. niloticus *from ten geographic sites across Africa (Table 1 and Figure 1). Our samplings aimed 1) to cover the macro-geographic area in the natural range of this species as extensive as possible and include different ichthyofaunal provinces and regions and therein hybrographic basins [30], and 2) to include successive temporal sampling through the short period of time in local populations, which allow us to survey the temporal genetic stability at the micro-geographic level. In both cases, a special attention was paid to avoid a potential influence of anthropogenic fish translocations and introductions, which could cause introgression and hybridisation. For that purpose, sampling sites without any record of *Oreochromis spp*. introduction were selected. In addition, all sampled individuals (considering only fully mature specimens, > 120 mm) were visually checked according to specific morphological characters [13] and confirmed the absence of sign of hybridisation for specimens analysed [16,42,43]. Sampling of Volta and Awash basins were done between 2001 and 2004, whereas samples from Lake Turkana, Lake Manzala, Niger and Senegal were a subset of populations already investigated for enzyme polymorphism and mtDNA [16,25,26], which have been considered pure *O. niloticus *at the nuclear genome level [16].

The sampling locations covered six hydrographic basins in Africa, belonging to the Sudano-Sahelian (Niger, Senegal and Volta) and Nilotic (Turkana, Nile) regions of the Nilo-Sudanian province and Ethiopian Rift valley (Awash basin) ichthyofaunal province [23,30,31] and represented four sub-species (*O. n. niloticus*, *O. n. vulcani*, *O. n. cancellatus *and *O. n. filoa*) according to their eco-morphological characters [13].

Analysis of micro-geographic and temporal variation was focussed on the Volta basin, where temporal sample series were collected repeatedly over a period of 18 months in two of the three geographic populations: Kpandu and Nyinuto (Table 1). These temporal samples were collected precisely at the same geographic location (verified by GPS coordinates) with the same standardised fishing protocol using seine nets. To minimise the potential impact of shoals' capture (*i.e*. biased sampling among relatives), each temporal sample was composed of individuals taken from multiple seine catches.

For our analyses, we considered two different datasets. (1) The macro-geographic analyses were conducted including all samples from the ten geographic populations considered (n = 350; temporal sample series from the two sites of the Volta basin pooled respectively into a single population-sample per site, *i.e*. Kpandu and Nyinuto). (2) The micro-geographic and temporal analyses were conducted at the scale of the entire Volta basin, including the samples from the 3 sites along the basin (n = 206): one sample from the Kou River (upstream Volta basin), four temporal samples from Kpandu (on the Lake Volta) and three temporal samples from Nyinuto (downstream on the Volta River) (Table 1 and Figure 1).

### Microsatellite typing

Nine unlinked microsatellites were selected from the *O. niloticus *genomic DNA library [53]. Genomic DNA was extracted from fin clips or muscle fragments stored in 95% ethanol using a phenol-chloroform procedure [54]. DNA samples were amplified by PCR using the indirect fluorescent tagging procedure described by Schuelke [55]. The amplification was performed using standard PCR-mix concentration and a touch-down PCR procedure, allowing standardised conditions over the loci analysed (see details in Additional file 1), using a 384-well-plate technology (Genotyping Platform, Genopole Montpellier Languedoc-Roussillon, CIRAD). PCR products were detected on an automated Li-Cor gel sequencer (IR2, Lincoln, Neb.), and genotyped were accessed by eyes.

### Genetic diversity Analysis

The total number of alleles (A), observed (*H*_o_) and non-biased expected (*H*_nb_) heterozygosities were calculated for each locus separately and across all loci using GENETIX 4.03 [56] and FSTAT 2.9.3 [57]. Allelic richness (Ar) was estimated for each population using rarefaction algorithm implemented in HP-rare [58], assuming an even number of genes per population, n = 20, based on the smallest population sample size in the study. For each sample, the fixation index, *F*_IS_, was estimated across all loci, and statistical significance was assessed with permutation tests (n = 1000) using GENETIX. Corrections for multiple tests were performed using the sequential Bonferroni procedure [59].

Heterozygote deficits, as indicated by *F*_IS _values significantly larger than 0, might reflect (1) sampling issues (*e.g*. Wahlund effect) or (2) impact of specific life history traits including a low dispersal or any social behaviour leading to a particular mating system. However, technical problems, such as null alleles, can also affect *F*_IS_. Thus, we first ran MICRO-CHECKER[60] to estimate if null alleles might affect the fixation index. Potential null alleles were identified at three loci (UNH142, UNH162, and UNH211), however, excluding these three loci had very minor effects on *F*_IS _(data not shown).

We used analysis of molecular variance, AMOVA [61], implemented in ARLEQUIN 2.0 [62] to test the potential population substructure due to different seine catches within the different temporal and local samples. We tested the possibility of non-random mating and inbreeding within population and cohorts as a potential cause of positive *F*_IS _values by calculating the degree of relatedness within samples using the pairwise relatedness index r_xy _of Queller & Goodnight [63] implemented in IDENTIX[64]. The observed arithmetic mean of r_xy _(Mr_xy_) in a sample was compared to that expected for a panmictic population. The observed variance of r_xy _(Vr_xy_) was also compared with the expectation from a panmictic population to test for the presence of multiple family groups within samples (which would increase the variance of r_xy_) [64,65]. For the sample pooled by station (Kp and Ny), the null distribution was generated by permuting genotypes within the population (1,000 permutations), following Castric *et al. *[65]. For the temporal samples, the null distribution was calculated based on 1,000 randomised sub-samples of the same size as the tested sample, taken from the total samples from the given geographic site (*i.e*. Kp or Ny) [66].

### Population clustering and genetic differentiation

Factorial correspondence analyses were performed using R [67] and the package ADE4 v1.4-9 [68] to investigate the relationship between populations from multi-locus genotypes without defining groups *a priori*. Successive correspondence analyses were performed to group populations from the largest to the finest geographic scales investigated.

Similarly, genetic population clustering was assessed using STRUCTURE v.2.3.1[32,33] and conducted at i) macro-geographic scale and ii) micro-geographic and temporal scales. Simulations were conducted assuming an admixture model, with 10,000 burn-in steps and 100,000 MCMC steps. Ten independent runs were performed for each number of clusters assumed (K), with K varying between 2 and z+1, with z being the number of samples including in the analysis (*i.e*. respectively 11 for macro-geographic and 9 for micro-geographic and temporal analysis). The optimal number of clusters was assessed based on correction proposed by Evanno et al. [34].

Genetic parameters, providing a measure of the genetic differentiation existing between two populations, can be calculated using different estimators (for a discussion see [69]). The *F*-statistics [70], based on the infinite alleles model (IAM), relies solely on allele identity information. As it is an easy target for homoplasy, leading to the underestimation of genetic differentiation among highly structured populations (where *μ *>*m*), this parameter generally is used to compare populations at small geographic scale. In contrast, the analogous *R*-statistics [71] based on the stepwise mutation model (SMM), takes into account allele size differences. As it typically suffers from high sampling variances, it is used for comparisons of large geographic scales. Here, as we were faced with distinct scales, we applied the test suggested by Hardy *et al. *[35] to choose the most suitable estimators. This test indicates whether or not allele sizes provide information on population differentiation, helping to choose between *F-*statistics and *R-*statistics in the most objective way. We applied this test implemented in SPAGeDi [72] for both datasets, and used the most pertinent statistics. Matrices of pairwise differentiation between populations (*R*-statistics or *F-*statistics) were computed using ARLEQUIN. The averaged genetic differentiation calculated over pairwise value between or within group of populations were noted $\bar{F_{\text{ST}}}$ or $\bar{R_{\text{ST}}}$.

Further, AMOVA [61] was also used to investigate how genetic variability was distributed in space and time. Considering macro-geographic scale, successive hierarchical levels were defined based alternatively on i) biogeographic regions and provinces (3 groups), ii) hydrographic basins (6 groups) and iii) sub-species taxonomy (4 groups - see Table 4 for details). Considering micro-geographic scales, hierarchical AMOVA was computed considering temporal samples within localities at i) the Volta basin and ii) the locality levels.

### Estimating effective population size and migration rate

We estimated effective population size (*N_e_*) and migration rate (*m*) based on the spatio-temporal samples from the Volta basin. In order to reduce the sampling biases due to the small sample size, we excluded a temporal sample with the smallest sample size (KpM2, n = 13) from the estimation, and then retained only larger samples (n ≥ 28). We used three different approaches to estimate *N_e _*(see discussion in [39,46,73]). Two of them are based on temporal variation of allele frequencies within populations. Both use likelihood methods and are implemented in MLNe 2.3 [36], but the first method (*N_e_*-_CLOSED_) assumes no immigration [38], assumption likely violated in the current context. The second method (*N_e_*-_open_), relaxes this assumption and allows the joint estimation of *N_e _*and *m *[36]. For these approaches, the three temporal samples were considered to be from successive generations, assuming a generation time of about 6 months, as generally accepted for the Nile tilapia [14]. For the estimation of *Ne*_open _of a given focal population, we pooled the samples from the other populations from the basin (*i.e*. Ko + Ny for Kp, and Ko + Kp for Ny) to mimic the allele frequencies in the source population of all potential immigrants. The third approach (LD-*N_e_*), is based on the linkage disequilibrium method [37] and implemented in NEESTIMATOR[74]. In this method, no migration is assumed. LD-*N_e _*was calculated for each temporal sample and then averaged over them. A point estimate and the 95% confidence intervals were obtained for each population by each method.

## Authors' contributions

EB, PB, XR, HA and JFB designed the research within the framework of the PhD project of EB; EB, YF, XR and AT collected the samples and data; EB and PB analysed the data; EB, PB, XR and HA led the writing. All authors read and approved the final manuscript.

## Acknowledgements

We would like to thank Eddie Abban and the team of the Water Research Institute (Ghana), Abebe Getahun (University of Addis Ababa), Pascal Bonnet & Géraud Laval (CIRAD/ILRI), the Office des Parcs Nationaux du Sénégal, Gabriele Hörstgen-Schwark (University of Göttingen), and Brendan McAndrew (University of Stirling) for their cooperation in obtaining samples. We also thank Stéphane Mauger, Gaëlle Chupot, René Guyomard (INRA), and Claire Billot (CIRAD) for their laboratory assistance; Agathe Bezault for the illustrations; Wendy Brand-Williams (INRA) for English improvement, and especially, Nicolas Poulet (ONEMA) for his help in statistical analyses and discussions about demography, Bernard Chevassus (INRA) and Mark Jobling (University of Leicester) for stimulating discussions, and Dylan Fraser (Dalhousie University), Irene Keller (EAWAG), Marta Barluenga (Museum of Natural History, Madrid), the editor Michael Hansen and the anonymous reviewers for their constructive comments. This study was supported by grants from the CIRAD, INRA & MENRT (France) to EB.

## Supplementary Material

Additional file 1 **Genotyping protocol**. Detailed microsatellite genotyping protocol. Click here for file

Additional file 2 **Genetic diversity information per loci and population**. Tables of genetic diversity estimators calculated per loci and population. Click here for file

Additional file 3 **Additional Structure Analyses**. Genetic Structure analysis at a) macro-geographic and b) micro-geographic and temporal scales, including plots of evolution of log-likelihood and individual cluster assignment for the different number of clusters K. Click here for file
