# Supplementary material for: Spatial and temporal variation in population genetic structure of wild Nile tilapia (Oreochromis niloticus) across Africa
Source: BMC Genet. 2011 Dec 9;12:102. doi: 10.1186/1471-2156-12-102 (PMC3260159; doi:10.1186/1471-2156-12-102)
Supplement: Additional file 1 — Genotyping protocol. Detailed microsatellite genotyping protocol. [file 1471-2156-12-102-S1.DOC]

# Spatial and temporal variation in population genetic structure of wild Nile tilapia (*Oreochromis niloticus*) across Africa

E. Bezault, P. Balaresque, A. Toguyeni, Y. Fermon,
H. Araki, J.F. Baroiller & X. Rognon

**Supplementary Material:**

**Additional file 1 - Detailed microsatellite genotyping protocol**

***Microsatellite source:*** Dinucleotides microsatellites from the *O. niloticus* genomic DNA library [1].

***DNA source:*** Genomic DNA extracted from fin or muscle fragments stored in 95% ethanol by phenol-chloroform protocol [2].

***PCR mix:*** DNA samples were amplified by PCR using the indirect fluorescent tagging procedure described by Schuelke [3]. The amplification was performed in a total volume of 10µl, containing 20ng DNA, 2mM MgCl_2_, 200µM dNTP, 0.08µM Forward-M13-tailed primer, 0.1µM of each Reverse and Universal-M13 primers, 1µl PCR buffer, 20ng/µl BSA, and 0.1U/µl Taq-polymerase.

***PCR program:*** A touch-down PCR procedure was applied to standardise the PCR conditions over all loci, allowing the simultaneous amplification of different loci within a plate (384-well plate technology), using a Mastercycler Eppendorf (Genotyping Platform, Genopole Montpellier-Languedoc-Rousillon, CIRAD, Montpellier).

The PCR program was as follows:

- initial denaturation at 94°C for 4’;

- 20 touch-down cycles, with denaturation at 94°C for 45”, annealing for 1’ starting at 58°C and decreasing by 0.5°C at each cycle, and elongation at 72°C for 1’15”;

- 20 cycles of amplification, with denaturation at 94°C for 45”, annealing at 48°C for 1’, and elongation at 72°C for 1’15”;

- and final elongation at 72°C for 5min.

***Electrophoresis and genotyping:*** PCR products were loaded on a 7% denaturing polyacrylamide gel and detected on an automated Li-Cor sequencer (IR2, Lincoln, Neb.). Determination of the genotypes was accessed by eyes based on the electrophoregrams.

**References**

1. Lee WJ, Kocher TD: **Microsatellites DNA markers for genetics mapping in *Oreochromis niloticus*.** *J Fish Biol* 1996, **49:**169-171.

2. Estoup A, Martin O: **Marqueurs microsatellites: Isolement à l'aide de sondes non-radioactives, caractérisation et mise au point**. 1996, [<http://www.agroparistech.fr/svs/genere/microsat/microsat.htm>].

3. Schuelke M: **An economic method for the fluorescent labeling of PCR fragments.** *Nature Biotechnology* 2000, **18:**233-234.
