# Supplementary material for: Spatial and temporal variation in population genetic structure of wild Nile tilapia (Oreochromis niloticus) across Africa
Source: BMC Genet. 2011 Dec 9;12:102. doi: 10.1186/1471-2156-12-102 (PMC3260159; doi:10.1186/1471-2156-12-102)
Supplement: Additional file 2 — Genetic diversity information per loci and population. Tables of genetic diversity estimators calculated per loci and population. [file 1471-2156-12-102-S2.DOC]

**Additional file 2 - Genetic diversity information per loci and population
Table A** Genetic diversity and allelic information for the nine loci and the ten populations analysed across Africa (sample codes are given in **Table 1**).

|  |  | | **Populations** | | | | | | | | | |
| --- | --- | --- | --- | --- | --- | --- | --- | --- | --- | --- | --- | --- |
| **Loci** |  | | Hr | Kk | Me | Mz | Tu | Se | Nb | Ko | Kp | Ny |
| **UNH-115** | | *A* | 5 | 3 | 3 | 5 | 8 | 4 | 3 | 6 | 8 | 8 |
|  | *H*_nb_ | | 0.693 | 0.508 | 0.499 | 0.807 | 0.821 | 0.540 | 0.468 | 0.774 | 0.736 | 0.632 |
|  | *H*_obs_ | | 0.640 | 0.690 | 0.577 | 0.786 | 0.733 | 0.400 | 0.400 | 0.875 | 0.646 | 0.653 |
| **UNH-129** | | *A* | 1 | 1 | 2 | 4 | 3 | 4 | 3 | 5 | 6 | 7 |
|  | *H*_nb_ | | 0.000 | 0.000 | 0.033 | 0.733 | 0.641 | 0.706 | 0.353 | 0.667 | 0.727 | 0.668 |
|  | *H*_obs_ | | 0.000 | 0.000 | 0.033 | 0.571 | 0.533 | 0.533 | 0.200 | 0.500 | 0.653 | 0.566 |
| **UNH-142** | | *A* | 2 | 2 | 3 | 4 | 3 | 8 | 5 | 6 | 10 | 7 |
|  | *H*_nb_ | | 0.427 | 0.035 | 0.426 | 0.611 | 0.536 | 0.885 | 0.668 | 0.722 | 0.726 | 0.694 |
|  | *H*_obs_ | | 0.400 | 0.035 | 0.391 | 0.500 | 0.600 | 0.933 | 0.800 | 0.688 | 0.558 | 0.714 |
| **UNH-146** | | *A* | 3 | 3 | 3 | 4 | 3 | 2 | 1 | 4 | 4 | 3 |
|  | *H*_nb_ | | 0.571 | 0.220 | 0.542 | 0.611 | 0.681 | 0.067 | 0.000 | 0.333 | 0.270 | 0.298 |
|  | *H*_obs_ | | 0.500 | 0.172 | 0.593 | 0.643 | 0.600 | 0.067 | 0.000 | 0.313 | 0.220 | 0.179 |
| **UNH-154** | | *A* | 2 | 2 | 2 | 5 | 6 | 5 | 5 | 4 | 6 | 4 |
|  | *H*_nb_ | | 0.171 | 0.503 | 0.508 | 0.725 | 0.789 | 0.775 | 0.768 | 0.413 | 0.533 | 0.457 |
|  | *H*_obs_ | | 0.111 | 0.464 | 0.607 | 0.750 | 0.600 | 0.733 | 0.700 | 0.500 | 0.534 | 0.329 |
| **UNH-162** | | *A* | 1 | 1 | 2 | 5 | 6 | 3 | 2 | 6 | 9 | 7 |
|  | *H*_nb_ | | 0.000 | 0.000 | 0.063 | 0.773 | 0.812 | 0.559 | 0.395 | 0.584 | 0.746 | 0.610 |
|  | *H*_obs_ | | 0.000 | 0.000 | 0.063 | 0.643 | 0.733 | 0.533 | 0.300 | 0.533 | 0.588 | 0.281 |
| **UNH-189** | | *A* | 4 | 3 | 4 | 8 | 6 | 10 | 2 | 10 | 14 | 13 |
|  | *H*_nb_ | | 0.554 | 0.194 | 0.592 | 0.878 | 0.853 | 0.897 | 0.190 | 0.831 | 0.882 | 0.872 |
|  | *H*_obs_ | | 0.519 | 0.138 | 0.429 | 0.857 | 0.800 | 0.933 | 0.200 | 0.750 | 0.639 | 0.701 |
| **UNH-211** | | *A* | 2 | 3 | 3 | 7 | 5 | 13 | 4 | 10 | 18 | 13 |
|  | *H*_nb_ | | 0.509 | 0.076 | 0.534 | 0.773 | 0.816 | 0.931 | 0.595 | 0.859 | 0.863 | 0.906 |
|  | *H*_obs_ | | 0.500 | 0.077 | 0.375 | 0.857 | 0.933 | 0.933 | 0.700 | 0.875 | 0.662 | 0.905 |
| **UNH-216** | | *A* | 1 | 1 | 1 | 3 | 4 | 4 | 2 | 6 | 10 | 7 |
|  | *H*_nb_ | | 0.000 | 0.000 | 0.000 | 0.569 | 0.680 | 0.693 | 0.337 | 0.653 | 0.757 | 0.656 |
|  | *H*_obs_ | | 0.000 | 0.000 | 0.000 | 0.308 | 0.643 | 0.643 | 0.200 | 0.688 | 0.755 | 0.603 |
| **Average** | | *A* | 2.333 | 2.111 | 2.778 | 5.000 | 5.333 | 7.444 | 3.000 | 6.333 | 9.889 | 8.556 |
|  | *H*_nb_ | | 0.325 | 0.171 | 0.363 | 0.720 | 0.736 | 0.672 | 0.419 | 0.648 | 0.693 | 0.644 |
|  | st. dev. | | ± 0.281 | ± 0.207 | ± 0.235 | ± 0.103 | ± 0.107 | ± 0.267 | ± 0.239 | ± 0.180 | ± 0.187 | ± 0.187 |
|  | *H*_obs_ | | 0.297 | 0.175 | 0.341 | 0.657 | 0.686 | 0.634 | 0.389 | 0.636 | 0.584 | 0.548 |
|  | st. dev. | | ± 0.264 | ± 0.243 | ± 0.247 | ± 0.180 | ± 0.126 | ± 0.291 | ± 0.280 | ± 0.189 | ± 0.151 | ± 0.237 |
|  | *F*_IS_ | | 0.09 | -0.03 | 0.06 | 0.09 | 0.07 | 0.06 | 0.08 | 0.02 | 0.16 | 0.15 |
| Bonferroni | | | ns | ns | ns | ns | ns | ns | ns | ns | *** | *** |

*A =* number of alleles observed per population; *H*_nb_ = non-biased expected heterozygosity; *H*_obs_ = observed heterozygosity; st. dev. = standard deviation; Bonferroni = significance of the *F*_IS_ after Bonferroni sequential correction: ns = non-significant, * *P* < 0.05, ** *P* < 0.01, *** *P* < 0.001.

**Table B** Genetic diversity at nine microsatellite loci in temporal sample series at spatio-temporal level whithin the Volta basin (sample codes are given in **Table 1**).

|  |  | | **Temporal samples** | | | | | | |
| --- | --- | --- | --- | --- | --- | --- | --- | --- | --- |
| **Loci** |  | | KpN1 | KpM2 | KpJ2 | KpF3 | NyN1 | NyM2 | NyF3 |
| **UNH-115b** | | *A* | 7 | 6 | 6 | 6 | 6 | 7 | 4 |
|  | *H*_nb_ | | 0.729 | 0.748 | 0.720 | 0.725 | 0.661 | 0.684 | 0.515 |
|  | *H*_obs_ | | 0.750 | 0.615 | 0.654 | 0.552 | 0.680 | 0.654 | 0.619 |
| **UNH-129** | | *A* | 6 | 6 | 6 | 6 | 7 | 5 | 5 |
|  | *H*_nb_ | | 0.685 | 0.810 | 0.730 | 0.682 | 0.767 | 0.543 | 0.609 |
|  | *H*_obs_ | | 0.519 | 0.636 | 0.714 | 0.724 | 0.667 | 0.423 | 0.615 |
| **UNH-142** | | *A* | 7 | 7 | 6 | 9 | 0 | 2 | 8 |
|  | *H*_nb_ | | 0.662 | 0.774 | 0.728 | 0.752 | - | 0.500 | 0.712 |
|  | *H*_obs_ | | 0.579 | 0.700 | 0.476 | 0.556 | - | 0.500 | 0.731 |
| **UNH-146** | | *A* | 3 | 3 | 3 | 3 | 5 | 3 | 2 |
|  | *H*_nb_ | | 0.105 | 0.342 | 0.320 | 0.331 | 0.546 | 0.198 | 0.035 |
|  | *H*_obs_ | | 0.036 | 0.385 | 0.300 | 0.241 | 0.296 | 0.214 | 0.035 |
| **UNH-154b** | | *A* | 3 | 4 | 5 | 6 | 2 | 3 | 3 |
|  | *H*_nb_ | | 0.437 | 0.68 | 0.422 | 0.620 | 0.396 | 0.471 | 0.492 |
|  | *H*_obs_ | | 0.533 | 0.615 | 0.400 | 0.633 | 0.143 | 0.310 | 0.483 |
| **UNH-162b** | | *A* | 4 | 2 | 7 | 6 | 6 | 5 | 5 |
|  | *H*_nb_ | | 0.652 | 0.500 | 0.579 | 0.809 | 0.758 | 0.463 | 0.665 |
|  | *H*_obs_ | | 0.667 | 0.500 | 0.429 | 0.679 | 0.500 | 0.207 | 0.280 |
| **UNH-189** | | *A* | 11 | 6 | 11 | 10 | 13 | 10 | 11 |
|  | *H*_nb_ | | 0.881 | 0.824 | 0.867 | 0.870 | 0.891 | 0.853 | 0.849 |
|  | *H*_obs_ | | 0.679 | 0.222 | 0.600 | 0.767 | 0.700 | 0.733 | 0.667 |
| **UNH-211** | | *A* | 9 | 3 | 12 | 14 | 0 | 0 | 13 |
|  | *H*_nb_ | | 0.856 | 0.833 | 0.841 | 0.891 | - | - | 0.906 |
|  | *H*_obs_ | | 0.520 | 1.000 | 0.621 | 0.889 | - | - | 0.905 |
| **UNH-216** | | *A* | 8 | 4 | 8 | 8 | 7 | 7 | 5 |
|  | *H*_nb_ | | 0.774 | 0.739 | 0.761 | 0.753 | 0.692 | 0.684 | 0.603 |
|  | *H*_obs_ | | 0.621 | 1.000 | 0.714 | 0.857 | 0.667 | 0.679 | 0.462 |
| **Average** | | *A* | 6.444 | 4.556 | 7.111 | 7.556 | 6.571 | 5.250 | 6.222 |
|  | *H*_nb_ | | 0.642 | 0.694 | 0.663 | 0.715 | 0.673 | 0.550 | 0.598 |
|  | st. dev. | | ± 0.240 | ± 0.167 | ± 0.186 | ± 0.167 | ± 0.162 | ± 0.196 | ± 0.253 |
|  | *H*_obs_ | | 0.545 | 0.630 | 0.545 | 0.655 | 0.522 | 0.465 | 0.533 |
|  | st. dev. | | ± 0.207 | ± 0.256 | ± 0.149 | ± 0.195 | ± 0.374 | ± 0.274 | ± 0.257 |
|  | *F*_IS_ | | 0.155 | 0.086 | 0.181 | 0.085 | 0.230 | 0.156 | 0.111 |
| Bonferroni | | | *** | ns | *** | * | *** | *** | * |

*A =* number of alleles observed per population; *H*_nb_ = non-biased expected heterozygosity; *H*_obs_ = observed heterozygosity; st. dev. = standard deviation; Bonferroni = significance of the *F*_IS_ after Bonferroni sequential correction: ns = non-significant, * *P* < 0.05, ** *P* < 0.01, *** *P* < 0.001.
