# Supplementary material for: Spatial and temporal variation in population genetic structure of wild Nile tilapia (Oreochromis niloticus) across Africa
Source: BMC Genet. 2011 Dec 9;12:102. doi: 10.1186/1471-2156-12-102 (PMC3260159; doi:10.1186/1471-2156-12-102)
Supplement: Additional file 3 — Additional Structure Analyses. Genetic Structure analysis at a) macro-geographic and b) micro-geographic and temporal scales, including plots of evolution of log-likelihood and individual cluster assignment for the different number of clusters K. [file 1471-2156-12-102-S3.DOC]

**Additional file 3 - Detailed population clustering analysis using Structure.**

Structure v.2.3.1 was used to assess the genetic population structure at both i) macro-geographic scale and ii) micro-geographic and temporal scales. We assumed an admixture model, with 10,000 burn-in steps followed by 100,000 MCMC steps. Ten independent runs were performed for each number of clusters assumed (K). K was computed from 2 to z+1, where z is the number of population-samples included in the analysis. The Log likelihood (lnP(D)) was recorded for each model, while the optimal number of clusters was assessed based on the procedure proposed by Evanno et al. (ΔK) .

Here, the evolution of LnP(D) and ΔK according to K are reported (**Fig A**), as well as the evolution of individual clustering for each K tested for both macro-geographic scale (**Fig B**), and micro-geographic and temporal scales analysis(**Fig C**).


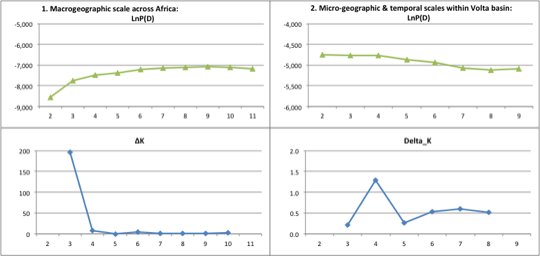


## Fig. A – Plot of log likelihood and ΔK of the calculated model in relation to the number of clusters assumed (K) for both macro-geographic scale and micro-geographic and temporal scales.


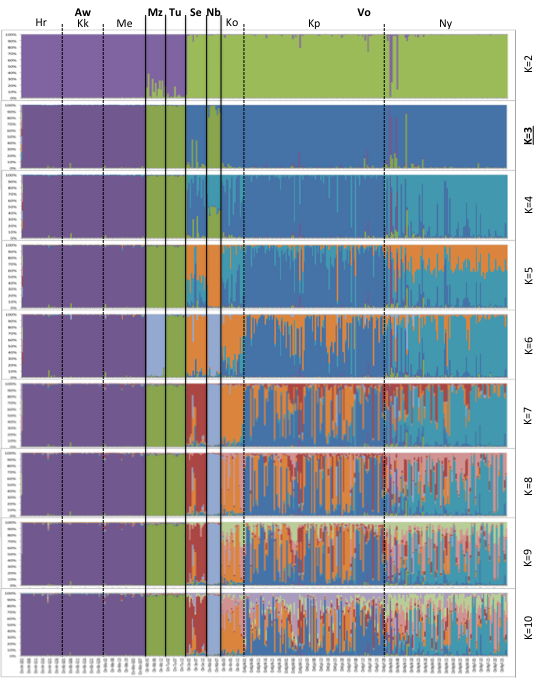


## Fig. B – Macro-geographic analysis: evolution of individual assignment with increasing number of assumed clusters, K = 2 - 10.


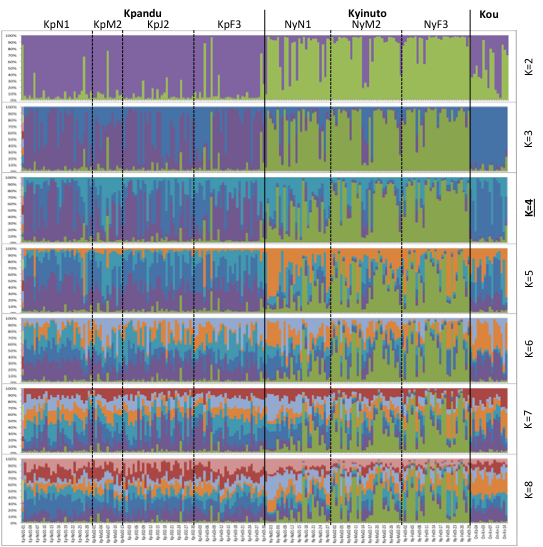


## Fig. C – Micro-geographic & temporal analysis: evolution of individual assignment with increasing number of assumed clusters, K = 2 - 8.
